# Recent Applications of Albumin as a Green and Versatile Catalyst in Organic Synthesis

**DOI:** 10.3390/molecules30214168

**Published:** 2025-10-23

**Authors:** Estefanía L. Borucki, Luis E. Iglesias

**Affiliations:** Laboratory of Biotransformations and Nucleic Acid Chemistry, National University of Quilmes, Roque Sáenz Peña 352, Bernal 1876, Buenos Aires, Argentina; estefania.borucki@unq.edu.ar

**Keywords:** biocatalysis, albumin, biocatalysts, catalytic promiscuity, green catalysis

## Abstract

Today, biocatalytic methodologies are well established as useful tools in green organic synthesis, since biocatalysts are mild, sustainable, and environmentally friendly catalysts which provide selectivity to the reactions they catalyse. Albumin, the most abundant protein of mammalian blood, is a versatile and mild biocatalyst in vitro. The aim of this review is to provide a perspective on the synthetic applications of albumin over the last decade. These cover transformations with a diverse chemical basis, such as additions, eliminations, and oxidations, including formation of carbon–carbon and carbon–heteroatom bonds. Albumin can also be applied in tandem and multicomponent reactions and offers a mild alternative for the synthesis of different heterocyclic cores. In addition to its synthetic possibilities, the remarkable reusability of this protein offers interesting potential from a biotechnological point of view.

## 1. Introduction

Today, it has become essential for chemical processes to be economical, safe, and environmentally friendly and to save on resources and energy. Very efficient and selective reactions are needed in order to obtain target products in high yields and minimize side products. Furthermore, most reactions are feasible in practice due to catalysis, but many catalysts involve drawbacks such as toxicity, environmental incompatibility, and harsh reaction conditions. Biocatalysis, the application of enzymes in the transformation of organic compounds [1,2], offers methodologies to overcome these challenges. Enzymes are sustainable and environmentally friendly catalysts with outstanding catalytic properties: they display regio-, chemo- and stereoselectivity, a feature minimizing side products; moreover, they operate under mild reaction conditions and have low energy requirements. Owing to these features, biocatalysis fulfils most of the green chemistry principles [1] and has remarkable potential for preventing environmental damage, as well as for saving on energy and non-renewable resources [3]. Thus, today, biocatalysts are established as green and useful tools in organic synthesis [4,5]; in addition, biocatalytic procedures are also applied by a wide variety of organic compound producers, such as the pharmaceutical and the food industries [6].

In addition to the features outlined above, enzymes possess a property which is crucial for their synthetic application: promiscuity. Enzymes do not really follow the old paradigm “one substrate, one enzyme, one activity”; they can accept non-canonical substrates even in non-aqueous medium. Moreover, enzymes exhibit catalytic promiscuity, defined as the ability of an enzyme to catalyze more than one type of transformation, i.e., a chemical transformation proceeding through a reaction mechanism different from that of the native transformation [7,8]. From a biochemical and evolutionary point of view, catalytic promiscuity is a phenomenon involving reactions that are physiologically irrelevant and differ from the transformation for which the enzyme has evolved [9,10]. However, promiscuous activities are latent, and the hidden catalytic machinery can demonstrate its capability when the enzyme is exposed to other substrates and environments [9,11]. This fact has tremendous potential in organic synthesis because it means enzymes can be repurposed for wider chemical transformations [3]; enabling catalytic promiscuity is considered an important challenge to broaden the utility of enzymes [12]. Furthermore, advances in protein engineering and directed evolution render possible the design of tailor-made enzymes according to the target chemical reaction [2,5].

Hydrolases, such as lipases, proteases, and acylases, are inexpensive enzymes and are well-known for their exquisite selectivity, which they achieve by hydrolysing and forming C-O and C-N bonds through nucleophilic acyl substitution [13]. In addition, they are emblematic examples of catalytically promiscuous enzymes displaying activity in C-C bond-forming reactions [14,15,16,17,18], which are among the most powerful organic reactions. Enzymes that build hydrocarbon backbones, such as aldolases, contrast with hydrolases because they are relatively expensive and usually possess narrow tolerance towards substrate structure; consequently, promiscuous hydrolases represent useful alternatives [19]. Even if mechanisms underlying catalytic promiscuity are far from being fully elucidated and continue to be matter of discussion [17,20], the ability of enzymes to catalyze unconventional reactions possesses great synthetic potential. Interestingly, promiscuity in broad sense is not a phenomenon restricted to enzymes but an inherent property of proteins [9,11]. This feature focuses on albumin, the target of this review.

Serum albumin, the most abundant protein of mammalian blood, is a water-soluble, globular, and monomeric protein. Its structure is composed of three alpha-helical homologous domains (I, II, and III), each of them containing two subdomains (A and B); serum albumins from different mammal sources are structurally conserved and have molecular weights of around 65–67 kDa [21]. In vivo albumin exerts transport functions: it binds a wide variety of exogenous and endogenous compounds, influencing their distribution and metabolism; in addition, it exerts a key role in the regulation of the osmotic pressure of blood. In contrast, this protein exhibits versatile catalytic activity in vitro, and today, it is considered a promiscuous biocatalyst [22]. The catalytic activity of bovine serum albumin (BSA) and human serum albumin (HSA) is ascribed to lysine residues of anomalous p*K_a_* (Lys-222 for BSA, Lys-199 for HSA), which are located in the hydrophobic binding site IIA of the protein [22]. BSA follows Michaelis–Menten kinetics in Kemp elimination of 5-nitrobenzisoxazole, and it has been shown that the involved catalytic base is Lys-222 [23,24,25]. Moreover, BSA-catalyzed aldol addition of acetone and 6-methoxy-2-naphthaldehyde also presents Michaelis–Menten kinetics [26], and this activity is conserved in a polypeptide derived from the albumin site IIA sequence [27]. On the other hand, as will be presented in this review, control experiments conducted with denatured albumin suggest that native structural integrity has an important role in the catalysis.

Regarding albumin sources (cow, human, rat, rabbit, pig, goat, and sheep), bovine albumin is available in high purity at low cost. This fact explains why BSA has been explored to a greater extent than albumin from other sources, including HSA [28]. BSA has been applied in transformations with a diverse chemical basis, such as redox, addition, condensation, and elimination reactions. Remarkably, as will be appreciated later, albumin is active in polar solvents such as ethanol and DMF. By the formation of C-C bonds, BSA has demonstrated catalytic activity in powerful synthetic reactions such as aldol addition, Knoevenagel, Henry, and Biginelli reactions [22].

From 2015 to the present, albumin has found further applications, and the aim of this short review is to update its potential as a green, mild, versatile, and easily available catalyst in organic synthesis. Single transformations catalyzed by albumin will be presented first (Section 2), followed by tandem and multicomponent reactions (Section 3); moreover, examples in the latter section will show the usefulness of albumin in heterocycle synthesis. On the other hand, examples illustrate the dramatic effect of reaction medium on albumin’s activity and selectivity, a feature shared by most biocatalysts. Due to the limited predictability of most suitable reaction conditions in biocatalysis (solvent, ratio of substrates, temperature, biocatalyst amount), the discussion of examples is based on the screening of experimental parameters, which is conducted to attain the most satisfactory results in terms of product yield and selectivity.

## 2. Single Transformations

### 2.1. C-C Bond and C-N Formation Reactions

#### 2.1.1. Cross-Aldol Condensation

**Figure 1:**
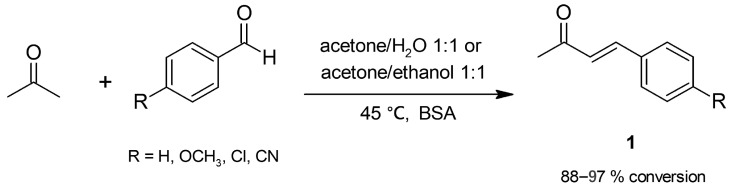
Bovine serum albumin (BSA)-catalyzed Claisen–Schmidt condensation of acetone.

Cross-aldol condensation is the well-known reaction of a carbonyl compound with an aldehyde in a strong basic medium; when an aromatic aldehyde is involved, it is known as the Claisen–Schmidt reaction. Initially, a β-hydroxycarbonyl compound (aldol) is formed by addition, whose subsequent elimination of water affords the target α,β-unsaturated carbonyl compound. The strong basic medium promotes the formation of double condensation products and Michael adducts as side products. In our laboratory, we have assayed BSA as a mild catalyst for Claisen–Schmidt reactions and first tested it in the condensation of acetone and benzaldehydes to give α,β-unsaturated carbonyl compounds (enones, **1**, Figure 1). The addition of water or ethanol as a cosolvent was crucial to obtain high conversions; moreover, a screening of reaction parameters (acetone/cosolvent ratio, BSA loading, and temperature) was conducted. In the absence of BSA, no reaction took place, excluding a non-catalyzed aldol condensation; furthermore, no reaction was observed in an assay carried out with denatured BSA, a fact suggesting that the native form of the protein is needed for catalytic activity. The most satisfactory conditions (Figure 1) allowed for high and specific conversions to enones; moreover, albumin demonstrated good potential for recyclability since no appreciable decrease in conversion to enones occurred after ten consecutive reaction cycles [29]. Experiments indicated that albumin catalyzes both the addition step and the dehydration of the intermediate β-hydroxyketone (ketol) to produce the enone, the latter being an activity not previously reported for BSA. This procedure represents a mild, green, and cheap alternative to the conventional Claisen–Schmidt reaction.

**Figure 2:**
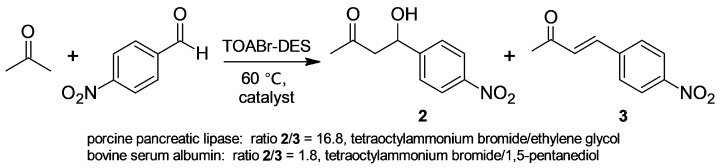
Reaction of acetone with *p*-nitrobenzaldehyde in tetraoctylammonium bromide–deep eutectic solvents (TOABr-DES) catalyzed by promiscuous catalysts.

In contrast, *m*- and *p*-nitrobenzaldehyde gave mixtures of the corresponding ketol and enone, which were only slightly enriched in the ketol. Computational experiments on BSA structural analysis and molecular docking of the assayed substrates were carried out in the zone of the BSA-binding site IIA containing Lys-222. Interestingly, only the nitrobenzaldehydes showed absence of contacts with the residues of the explored cavity [29]. Holtmann and coworkers [30] studied the reaction of acetone and *p*-nitrobenzaldehyde catalyzed by promiscuous biocatalysts in deep eutectic solvents (DES) at different temperatures (Figure 2). In the assayed DES (choline chloride/glycerol, tetraoctylammonium bromide (TOABr)/ethylene glycol (EG), and TOABr/1,5-pentanediol (PD)), BSA did not allow for selective catalysis; in both TOABr-DES, it led to mixtures of ketol **2** and enone **3**, which were again only slightly enriched in the ketol. However, in TOABr/EG, porcine pancreatic lipase (PPL) catalyzed the highly selective formation of ketol **2**, exhibiting selectivity complementary to BSA.

We have also assayed albumins from other sources (human, rat, rabbit, and pig) in the reaction of acetone and *p*-formylbenzonitrile (Figure 1); all tested albumins were active in the cross-aldol condensation. Structural and evolutionary analysis of the albumins suggested that the key residues involved in the catalysis are not only anomalous lysines but also a couple of basic residues possessing abnormal p*K_a_* values (Arg-199 and Lys-222 for BSA, Lys-199 and Arg-222 for HSA, and Arg-199 and Arg-222 for rabbit serum albumin). Such residues are conserved in the hydrophobic binding site IIA of the protein, an evolutionary conserved cavity connected with the protein surface through an also-conserved tunnel [31]. These results offer further evidence supporting albumin’s specific catalysis.

**Figure 3:**
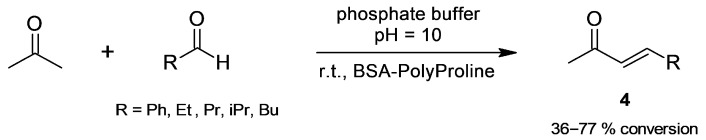
Cross-aldol condensation of acetone catalyzed by modified BSA.

Recently, Zhang and coworkers [32] applied transfer radical polymerization to prepare a proline-based conjugate from BSA and grafted a polymer bearing L-proline moieties onto the surface of albumin. The conjugate (BSA–PolyProline) was assayed at pH 10 and room temperature in the cross-aldol condensation of acetone and a set of aldehydes (Figure 3); under these conditions, enones **4** were selectively produced in high enone/ketol ratios and at a 36–77% conversion. The authors argue that the locally concentrated L-proline moieties surrounding the BSA molecule switch its catalytic mechanism, producing a reversal of the selectivity observed with free proline–BSA. In addition, this artificial enzyme was coupled to an ene-reductase to produce aliphatic ketones from acetone and aldehydes via tandem catalysis.

**Figure 4:**
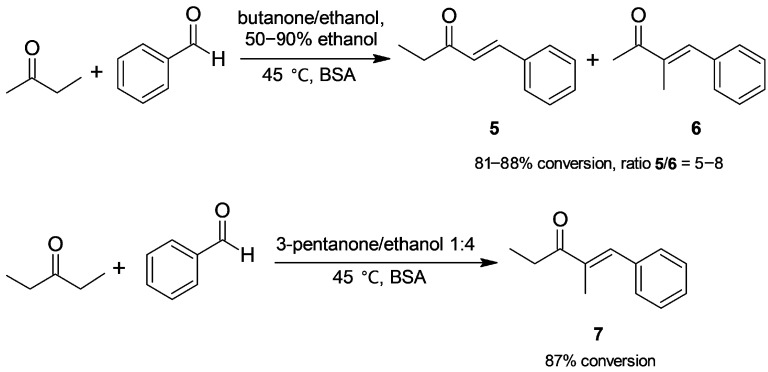
BSA-catalyzed Claisen–Schmidt condensation of butanone and 3-pentanone.

Apart from acetone, we have tested the BSA-catalyzed Claisen–Schmidt condensation of a set of ketones. By assaying butanone (Figure 4), the reaction medium was chosen through a screening of binary and tertiary mixtures [33]. Compared to BSA-catalyzed condensations of acetone (Figure 1), water gave low reactivity. The most satisfactory results were found in butanone/ethanol mixtures enriched in the latter (50–90%), which led to highly selective conversion to enones **5** and **6** and a low content of ketols (<5% conversion). Enones were obtained with slight regioselectivity towards the thermodynamically less stable product, formed from the less hindered α-carbon of butanone carbonyl; this suggests the role of steric factors in BSA catalysis. 3-Pentanone afforded the only expected enone **7** in an 87% conversion, free of intermediate ketols.

**Figure 5:**
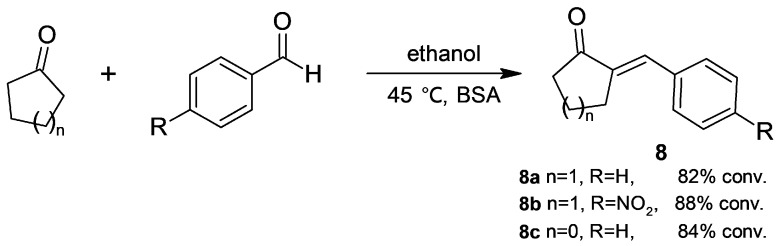
BSA-catalyzed Claisen–Schmidt condensation of cyclic ketones.

By testing nonvolatile cyclic ketones (Figure 5), the molar ratio of substrates was assayed in order to find the lowest suitable ketone concentration. Enone **8a** was obtained in a high conversion and free from ketols at a low excess of cyclohexanone (benzaldehyde/cyclohexanone molar ratio = 2); cyclopentanone exhibited a similar behaviour. In contrast, when *p*-nitrobenzaldehyde was assayed, cyclohexanone gave high and selective conversion towards enone **8b**, while cyclopentanone afforded a mixture of ketols and enone **8c** with low selectivity. In addition, kinetic data of the BSA-catalyzed reaction of cyclohexanone and *p*-nitrobenzaldehyde suggests an ordered bi-bi mechanism for enone formation [33].

#### 2.1.2. Henry Reaction

**Figure 6:**
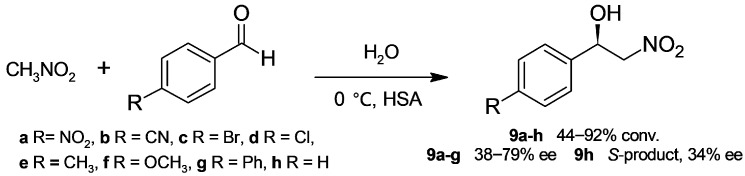
1-Phenyl-2-nitroalcohols obtained through human serum albumin (HSA)-catalyzed Henry reaction.

Albumin has been applied to catalyze the nitroaldol reaction (known as the Henry reaction), which affords synthetic useful β-nitroalcohols. Matsumoto and coworkers [34] assayed the reaction of nitromethane and *p*-nitrobenzaldehyde in water as a green solvent and tested albumins from different sources (bovine, chicken egg, goat, rabbit, porcine, and human). Even if high conversions to β-nitroalcohol **9a** were reached, the reactions were not enantioselectivem except for HSA; denatured HSA did not afford an enantioselective reaction, indicating the role of protein conformational integrity in stereoselectivity. HSA catalysis was extended to a set of substituted benzaldehydes at low temperature (Figure 6), which improved stereoselectivity. Moderate enantioselectivities towards *R*-products **9a**–**g** were reached; unexpectedly, the reaction of benzaldehyde proceeded with the opposite stereoselectivity (**9h**).

**Figure 7:**
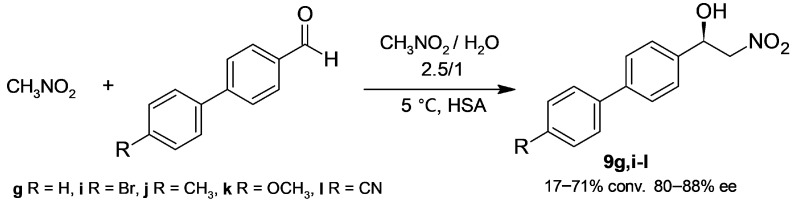
1-Biphenyl-2-nitroalcohols obtained through human serum albumin (HSA)-catalyzed Henry reaction.

The same group extended HSA catalysis to a set of biphenyl aldehydes and studied the effect on enantioselectivity of temperature, substrate ratio, and nitromethane/water ratio [35]; under the most satisfactory conditions (Figure 7), products **9g**,**i**–**l** were obtained in 80–88% ee. In addition, β-nitroalcohols **9a** and **9g** were prepared at the gram scale in good yields. On the other hand, the position of the phenyl substituent of benzaldehyde strongly affected the reactivity and stereoselectivity: 4-phenylbenzaldehyde gave a 71% conversion to **9g** in 85% ee, but 2-phenylbenzaldehyde produced a 33% conversion to the corresponding *R*-nitroalcohol in 35% ee, while no reaction was observed with 3-phenylbenzaldehyde.

#### 2.1.3. Knoevenagel Condensation

The Knoevenagel condensation is a variation of the aldol condensation involving nucleophilic addition of an aldehyde or a ketone to a compound bearing an activated methylene. Depending on the structure of the latter, this reaction typically affords α,β-unsaturated carbonyl compounds or α,β-unsaturated nitriles, which can undergo a subsequent Michael addition in the basic reaction medium. Consequently, green alternatives avoiding side reactions and harmful reagents are valuable methodologies; today, procedures based on the catalytic promiscuity of lipases are considered useful tools for the Knoevenagel condensation [16,36,37].

**Figure 8:**
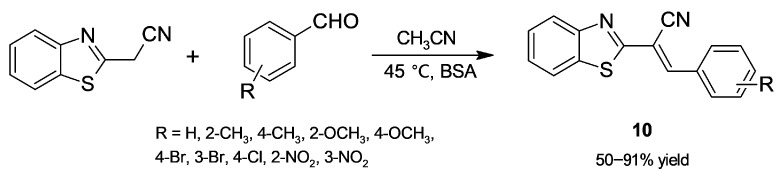
Benzothiazole-2-acetonitrile derivatives obtained by bovine serum albumin (BSA)-catalyzed Knoevenagel condensation.

Regarding albumin, Fedosov and coworkers [38] undertook a study to corroborate lipase’s promiscuous activity in Knoevenagel condensations and also assayed BSA. These authors found that it is active in the condensation of malononitrile with benzaldehyde at 25 °C in anhydrous ethanol to give benzylidenemalononitrile, although assayed lipases gave faster catalysis. As will be presented later (Section 3.2), the ability of albumin to recognise malononitrile as a substrate for Knoevenagel condensations is key to building many heterocyclic cores. On the other hand, Habibi and coworkers [39] have recently studied the reaction between benzothiazole-2-acetonitrile and benzaldehydes (Figure 8). Assayed biocatalysts were BSA and lipase B from *Candida antarctica* (CALB), a robust and versatile biocatalyst [13,14,15,16,17]; in contrast to albumin, poor results were obtained with CALB. It is worth mentioning that products **10** were submitted to a subsequent Michael addition with β-ketoesters; inversely, in this case only CALB afforded satisfactory yields. These results also illustrate the limited predictability by choosing a biocatalyst and the need for screenings in biocatalyzed reactions.

#### 2.1.4. Imine Formation

**Figure 9:**
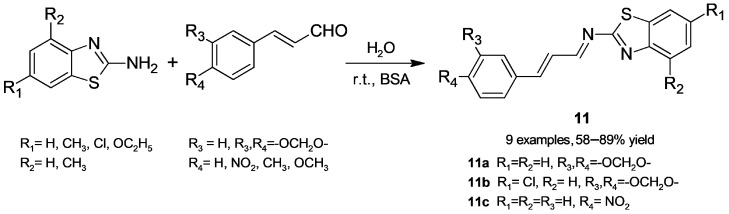
Schiff bases from 2-aminobenzothiazoles and cinnamaldehydes obtained by BSA catalysis.

Imines, condensation products resulting from the addition of a primary amine to a carbonyl compound with subsequent elimination of water, are useful precursors for amines, and their synthesis is usually carried out at controlled acid pH. Looking for a mild procedure to obtain imines (**11**, Figure 9) from 2-aminobenzothiazoles and cinnamaldehydes, Sinha, Nazir, and coworkers [40] found that BSA in water at room temperature is a useful catalyst. Albumins from other sources (rabbit, pig, sheep, and chicken) were less effective; imines (Schiff bases) were obtained in a 58–89% yield using a mild and green protocol. These products were assayed in a *C. elegans* model of Alzheimer’s disease, and **11a**–**c** showed potential as antiamyloid agents. This method was also extended to some anilines, providing the target Schiff bases in satisfactory yields.

### 2.2. Oxidations

#### 2.2.1. Oxidation of Thiols into Disulfides

**Figure 10:**
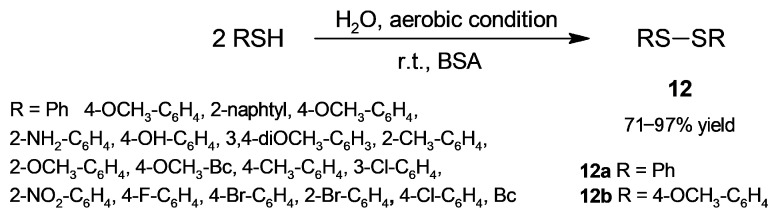
BSA-catalyzed oxidation of thiols into disulfides.

Based on BSA catalysis, Sinha and coworkers [41] developed a protocol for the conversion of thiols into disulfides (Figure 10), an activity not previously reported for albumin. The reaction was carried out under aerobic conditions in water at room temperature and tested first with thiophenol. BSA catalysed the formation of diphenyl disulfide (**12a**) in a 75% yield, while albumins from other sources (rabbit, sheep, egg, and chicken) afforded poor yields; moreover, the assayed lipases gave no reaction. Reactions carried out without BSA under aerobic conditions and with BSA in degassed water under a nitrogen atmosphere did not afford the product, while reaction with BSA in water under a nitrogen atmosphere produced 37% disulfide. These experiments indicate the crucial role of BSA and aerial oxygen in the target reaction. The scope of the oxidation was extended to a set of different substituted thiophenols (Figure 10), which afforded the corresponding disulfides in a 71–97% yield. In addition, a set of different heterocyclic thiols (pyridine, pyrimidine, thiazole, benzothiazole, benzoxazole, and thiadiazole cores) gave disulfides in a 40–90% yield. Among the assayed aliphatic thiols (allylthiol, cyclohexanethiol, and cysteine), only allylthiol was converted into disulfide in a good yield (78%). On the other hand, the reaction of *p*-methoxybenzenethiol was assayed for BSA recyclability, and after four reaction cycles, the **12b** yield only decreased from 97% to 90%. In addition, disulfide **12b** was prepared at the gram scale in a 92% yield. In addition to its mildness, the advantages of this oxidative procedure are the use of a green oxidant (air), a green solvent (water), and its waste-free nature.

#### 2.2.2. Sulfoxidation

**Figure 11:**
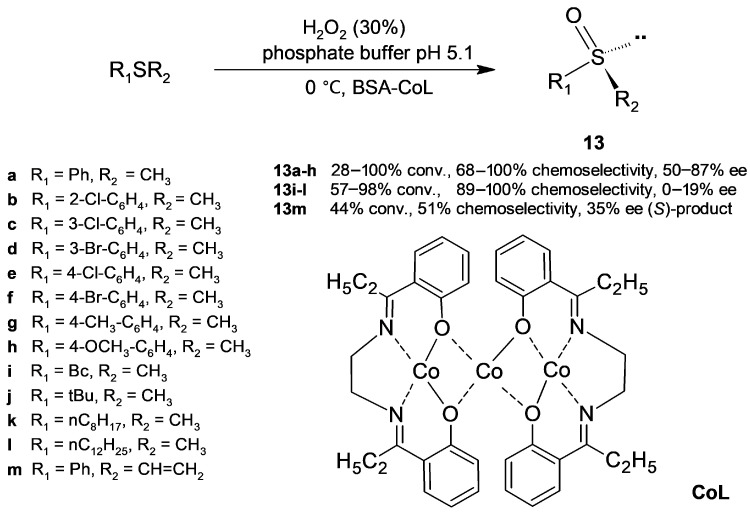
Oxidation of sulfides into sulfoxides catalyzed by the metalloenzyme mimic BSA (bovine serum albumin)-CoL.

Artificial metalloenzymes result from the incorporation of a metal complex with catalytic activity into a macromolecular host, such as a protein, by covalent or coordination binding as well as by supramolecular anchoring [42]. In the latter case, the host protein should be resistant to denaturation, soluble in water, and commercially available at a reasonable cost; consequently, albumin is an attractive candidate to design artificial metalloenzymes. Broadening the application of metalloenzyme mimics based on albumin to oxidise sulfides into sulfoxides [22], Bian, Liang, and coworkers [43] introduced a cobalt (II)–Schiff base complex into BSA and assayed the resulting hybrid biocatalyst (BSA-CoL) in the reaction of sulfides with hydrogen peroxide (Figure 11). To optimize the reaction parameters (pH, concentration of substrate, oxidant and catalyst, and temperature), thioanisole was chosen as a model substrate; the most satisfactory conditions (Figure 11) led to 98% conversion into sulfoxide **13a** (50% ee), which was free of sulfone (100% chemoselectivity). A blank reaction in the absence of the hybrid biocatalyst only produced a low conversion to a mixture of racemic sulfoxide and sulfone; in addition, non-hybrid BSA gave a very high conversion (85%) and 100% chemoselectivity towards sulfoxide but lower enantioselectivity (8% ee). Most of the sulfides were obtained in a good conversion and chemoselectivity through this mild procedure in aqueous medium involving a green oxidant (molar ratio of hydrogen peroxide/sulfide = 1.5). The oxidation of the aliphatic sulfides **13j**–**l** was highly chemoselective but proceeded with low (**13k**) or null (**13j**,**l**) stereoselectivity, while oxidation of phenyl vinyl sulfide showed reverse enantioselectivity (**13m**).

Huang, Bian, and coworkers [44] extended the above approach using the same ligand and prepared further metal (Cu (II), Mn (III), V (IV), and Fe (III))–Schiff base complexes, which were incorporated into albumins from different sources (bovine, human, porcine, rabbit, and sheep). The obtained biocatalysts were tested in the oxidation of sulfides **13a**–**i**,**m**, and the best results in terms of conversion and enantioselectivity were reached with hybrid biocatalysts from HSA; with vanadium (IV)–Schiff base–HSA at pH 5.1 and room temperature, eight of the ten tested sulfides afforded sulfoxides in a 93–100% conversion. Sulfoxide **13m** gave the highest enantioselectivity (94% ee of the *S*-enantiomer); based on the UV–vis spectra of the metalloenzyme mimics, it was concluded that stronger binding between complex and albumin led to higher enantioselectivity. Kinetic data suggested that the rate-limiting step is the interaction between the complex and hydrogen peroxide to form the active species, hypothetically, a metal peroxide complex.

**Figure 12:**
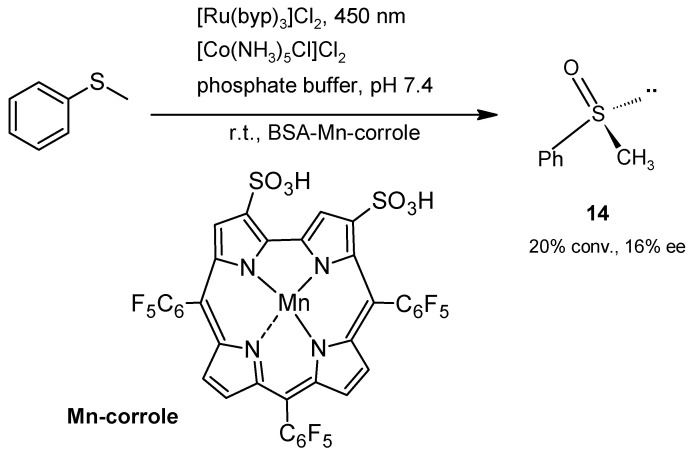
Light-induced oxidation of thioanisole catalyzed by the metalloenzyme mimic BSA-Mn-corrole.

On the other hand, Gross, Banse, Mahy, and coworkers [45] designed a metalloenzyme by incorporating a Mn (III)-sulfonated corrole (Figure 12) into BSA and assayed the hybrid in vis-light-induced oxidation of thioanisole at pH 7.4, with a ruthenium (II)–trisbipyridyl complex as a photosensitizer and a cobalt (III) complex as an electron acceptor. Even if the conversion and enantioselectivity were modest, this is a mild and green procedure for oxidation that avoids the use of an external oxidizing agent, since it involves oxygen transfer from water by the corrole attached to BSA.

**Figure 13:**
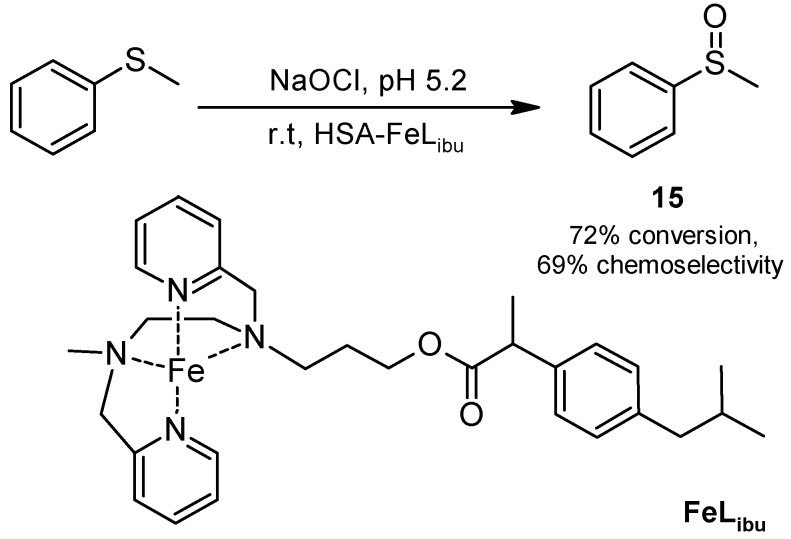
Oxidation of thioanisole catalyzed by the metalloenzyme mimic HSA-FeL_ibu_.

HSA has several binding sites for drugs that have been identified and structurally characterized [46,47]. Exploiting this fact, Ménage and coworkers [48] explored an alternative approach and synthesized a ligand derived from ibuprofen, complexed it with iron (II), and incorporated the resulting complex into HSA (Figure 13). The resulting biocatalyst was assayed in the oxidation of thioanisole using sodium hypochlorite at pH 5.2 and room temperature. The reaction gave a 72% conversion into racemic sulfoxide **15** with 69% chemoselectivity; conversion only decreased 8% after four reaction runs, a fact demonstrating the stability of the hybrid.

#### 2.2.3. Epoxidation

**Figure 14:**
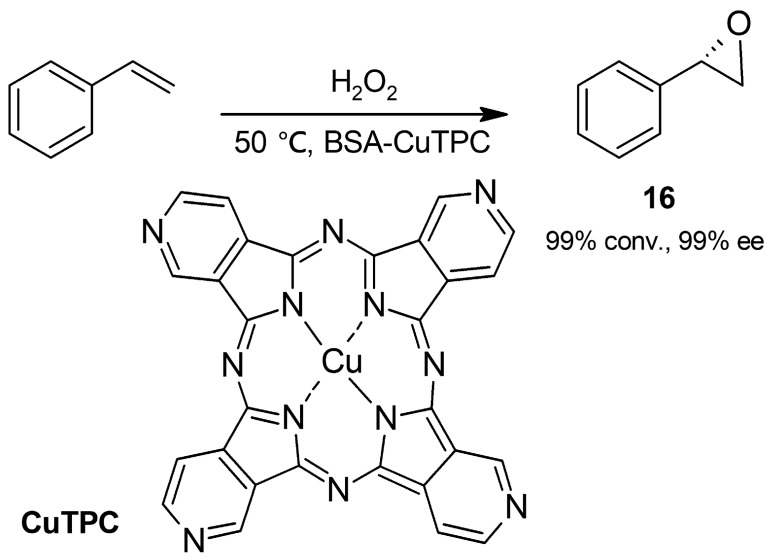
Oxidation of styrene into (*R*)-styrene oxide catalyzed by the metalloenzyme mimic BSA (bovine serum albumin)-CuTPC.

Recently, albumin as a metalloenzyme mimic has also been explored to catalyze the highly enantioselective oxidation of alkenes into epoxides. He and coworkers [49] have recently incorporated a synthetic complex of copper and phthalocyanine (CuTPC, Figure 14) into BSA and tested the resulting hybrid in the reaction of styrene with hydrogen peroxide. The biotransformation was conducted at 50 °C in a microemulsion of cetyltrimethylammonium bromide (CTAB) to solubilize styrene, and a 99% conversion towards the (*R*)-epoxide **16** (99% ee) was obtained; in addition, the epoxidation followed Michaelis–Menten kinetics. Complex-free BSA afforded epoxide in high stereoselectivity but allowed only very low substrate conversion, while denatured BSA gave no stereoselective reaction. Both CuTPC in the absence of BSA and denatured hybrid CuTPC-BSA gave considerably less satisfactory results, concluding that the high stereoselectivity attained with the hybrid arises from the chiral environment provided for CuTPC by BSA. In addition to peroxidase activity, the hybrid immobilized on an electrode displayed oxidase activity and catalyzed the conversion of oxygen into hydrogen peroxide through electrocatalytic cycles of Cu (I)/Cu (II). The metalloenzyme mimic was then applied for hydrogen peroxide generation and subsequent efficient one-pot epoxidation of styrene. In this way, the hybrid offered a green procedure for highly enantioselective epoxidation using either oxygen or hydrogen peroxide as oxidants.

## 3. Tandem and Multicomponent Reactions

Tandem and multicomponent reactions (MCRs) are useful processes since tandem reactions replace multi-pot stepwise processes by one-pot reactions, while MCRs combine three or more compounds to afford the target product. Therefore, they avoid intermediate workup steps and usually involve atom efficiency. The following examples covering the last decade illustrate the potential of albumin in these processes.

### 3.1. C-S Bond Formation Reactions

**Figure 15:**
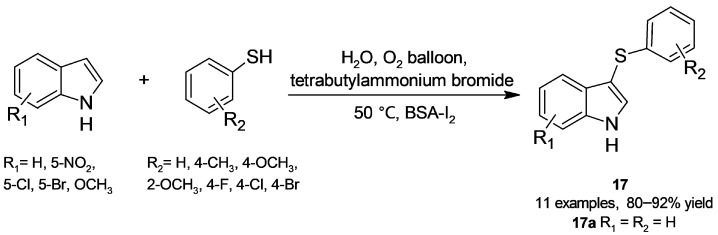
BSA-I_2_-catalyzed sulfenylation of indoles.

Sinha and coworkers have explored albumin as a mild and green alternative to form C-S bonds. On the one hand, they focused on the synthesis of 3-sulfenylindoles, which possess interesting biological activity [50]; exploiting their already-discussed BSA-catalyzed access to disulfides [41] (Section 2.2.1), they reported a metal- and base-free methodology to obtain 3-sulfenylindoles (**17**, Figure 15) [51]. Key to the strategy is the combination of BSA and iodine as a cooperative catalyst; the study of the reaction between indole and thiophenol comprised a screening of parameters such as albumin source (bovine, pig, sheep, rabbit), iodine amount, and phase transfer catalyst (the most satisfactory results were found with tetrabutylammonium bromide, TBAB). It is worth noting that no reaction was observed when assaying CALB catalysis. In addition to the structures shown in Figure 15, good high product yields were also obtained when assaying heterocyclic thiols as well as 1-methyl- and 2-methylindole. On the whole, advantages of the procedure are the aqueous medium, oxygen as the oxidizing agent, good substrate scope, and satisfactory product yields. The authors propose that albumin initially catalyzes the oxidation of thiophenol into disulfide [41], which subsequently reacts with iodine to produce phenylsulfenyl iodide (PhSI). This electrophilic species, detected by ESI-MS analysis of reaction aliquots, accomplishes indole sulfenylation with concomitant hydriodic acid formation; finally, reaction of HI with oxygen regenerates iodine. Addition of a radical scavenger such as TEMPO or BHT did not affect the **17a** yield, supporting an ionic rather than a radical mechanism. Moreover, the procedure was extended to produce a set of sulfenylated derivatives from 2-naphthol and 4-hydroxycoumarin.

**Figure 16:**
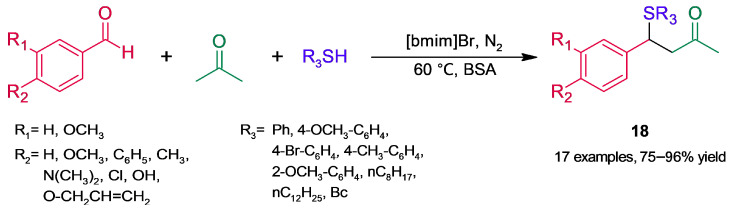
BSA-catalyzed synthesis of β-aryl-β-sulfidocarbonyl compounds in ionic liquid.

On the other hand, the same research group developed a strategy to obtain β-aryl-β-sulfidocarbonyl compounds (**18**, Figure 16) in good high yields based on a BSA-catalyzed three-component reaction in ionic liquid (IL) [52]. The mild and base-free procedure involves tandem aldol condensation–thia-Michael addition; in the former reaction, a benzaldehyde and acetone react to give an enone, whose reaction with a thiol affords the target products. In contrast with the synthesis of sulfenylated indoles discussed above, the biotransformations were conducted under a nitrogen atmosphere to avoid transformation of nucleophilic thiol into disulfide. The most satisfactory reaction conditions were found after screening for temperature, thiophenol amount, biocatalyst (albumin loading and source), and IL. By testing either BSA in the absence of 1-butyl-3-methylimidazolium bromide ([bmim]Br) or IL in the absence of BSA, the initial aldol condensation was not even observed, a fact suggesting a synergistic interaction between BSA and IL. To gain insight into the synergism, the procedure was studied by NMR and DFT, and a mechanism for the tandem reactions was proposed, based on a lysine residue of BSA. In addition, the catalytic system showed good recyclability up to five runs.

### 3.2. Heterocycle Synthesis

Heterocyclic compounds comprise a myriad of structures displaying a huge scope of pharmacological activities. Many well-known methods to build heterocyclic cores involve harsh reaction conditions (strong acid or basic medium, high temperature) that promote side reactions and lead to low yields; consequently, finding mild and green alternatives for heterocycle synthesis is an active area of research [53,54]. Biocatalysis offers mild, efficient, and environmentally friendly alternatives to build heterocyclic cores [55,56]. Focusing on catalytic promiscuity, enzymes such as lipases, amylases, and laccases are useful biocatalysts to synthesize heterocyclic cores [57,58]; on the other hand, promiscuous enzymes can also be successfully employed in MCR, leading to heterocycles [59]. Many heterocyclic rings are constructed through tandem reactions involving Knoevenagel condensation as one of the steps [60], so that the already-discussed BSA activity in Knoevenagel reactions (Section 2.1.3) suggests the potential of albumin in heterocycle synthesis. BSA has already been reviewed to build coumarins, 2-aminothiophenes, and 3,4-dihydropyrimidin-2-(1*H*)-one cores [22]. Over the last decade, albumin has expanded its applications as a biocatalyst for heterocycle synthesis.

#### 3.2.1. Oxygenated Heterocycles

**Figure 17:**
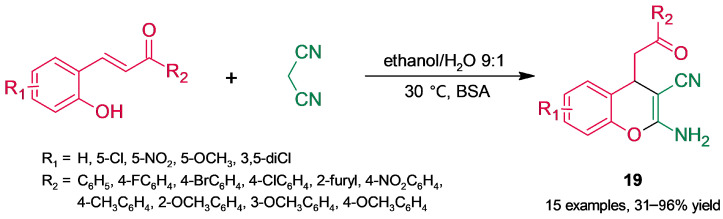
BSA-catalyzed synthesis of 2-amino-3-cyano-4*H*-chromenes.

4*H*-Chromenes possess strong cytotoxicity against several human cancer cell lines; in particular, compounds bearing 2-amino- and 3-cyano substitution have received attention [61]. Guan, He, and coworkers [62] reported the synthesis of 2-amino-3-cyano-4*H*-chromene derivatives (**19**) through a mild BSA-catalyzed tandem Michael addition–cyclization followed by tautomerization (Figure 17). Optimization of the biotransformation was carried out using 2-hydroxychalcone as a model substrate and involved a wide screening of reaction parameters (solvent, molar ratio of the substrates, water content, BSA loading, and temperature), most of them strongly affecting the reaction yield. In addition, no reaction occurred in the absence of BSA, while assays with denatured BSA gave poor results. This offers further evidence supporting the role of native structural integrity in catalytic activity.

**Figure 18:**
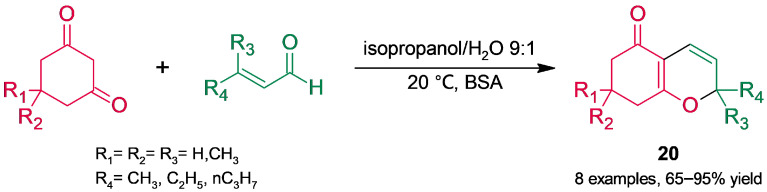
BSA-catalyzed synthesis of 7,8-dihydro-2*H*-chromen-5(6*H*)-ones.

Zhou and coworkers [63] used BSA catalysis to obtain a skeleton derived from 2*H*-chromen-5(6*H*)-one (Figure 18) through a tandem process involving 1,2-addition of the dione to the α,β-unsaturated aldehyde, intramolecular addition, and water elimination. Among the assayed reaction parameters (solvent, molar ratio of the substrates, water content, BSA loading, and temperature), temperature had no significant impact on conversion. In isopropanol–water at 9:1 and at 20 °C, a set of 7,8-dihydro-2*H*-chromen-5(6*H*)-ones (20) were produced in moderate–high yields.

**Figure 19:**
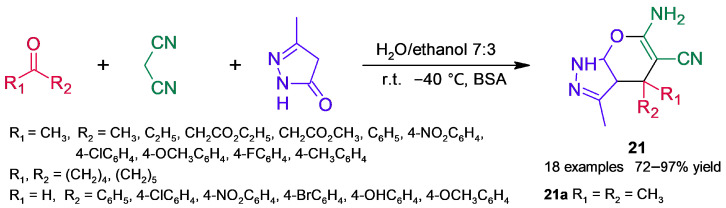
BSA-catalyzed MCR to obtain dihydropyrano[2,3-*c*]pyrazoles.

Pyrano[2,3-*c*]pyrazole scaffold exhibits diverse biological activity; consequently, interest in the green synthesis of this core and its derivatives has increased over the last decade [64]. Chaudhari and coworkers [65] studied the biocatalyzed three-component reaction of acetone, malononitrile, and 3-methyl-1*H*-pyrazol-5-(4*H*)-one (Figure 19), which occurred through tandem Knoevenagel condensation–Michael addition followed by cyclization and tautomerization. Substrates were assayed in an equimolar ratio, and since BSA surpassed the assayed promiscuous enzymes (lipases, proteases, and α-amylase), it was chosen for further optimization of reaction parameters (solvent, BSA loading, and temperature); among them, solvent and water content had a drastic effect on the product yield. Under the most satisfactory conditions (Figure 19), the corresponding dihydropyrano[2,3-*c*]pyrazole **21a** was obtained in a 94% yield, and the procedure was extended to a set of ketones and aldehydes, obtaining good-to-high product yields. An assay of isatines as carbonyl compounds resulted in obtaining spiro[indoline-3,4′-pyrano[2,3-*c*]pyrazole]derivatives in excellent yields. In addition, the procedure was carried out at the gram scale and by testing BSA recyclability, and only a slight decrease in product yield was observed after four reaction cycles. A mechanism for the MCR synthesis was proposed based on a lysine residue of BSA.

**Figure 20:**
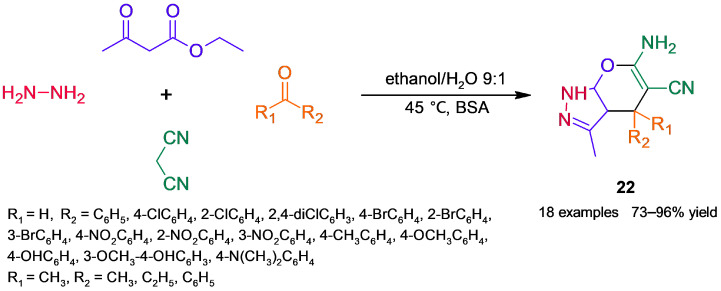
BSA-catalyzed four-component reaction to obtain dihydropyrano[2,3-*c*]pyrazoles.

In another BSA-catalyzed approach to dihydropyrano[2,3-*c*]pyrazoles **22** (Figure 20), Li and coworkers [66] built both heterocyclic moieties of the target products by exploring a four-component reaction between hydrazine hydrate, malononitrile, ethyl acetoacetate, and several carbonyl compounds, tested at an equimolecular ratio (Figure 20). Compared to the tandem process presented above, this MCR adds a step to form 3-methyl-1*H*-pyrazol-5-(4*H*)-one by condensation of ethyl acetoacetate and hydrazine, which subsequently react by the tandem reactions mentioned above; a mechanism based on a lysine residue of BSA was proposed. BSA again outperformed the other assayed biocatalysts and showed only a slight loss of activity after five reaction cycles; on the other hand, by screening reaction parameters, the survey of reaction solvents was crucial to reach high product yields.

**Figure 21:**
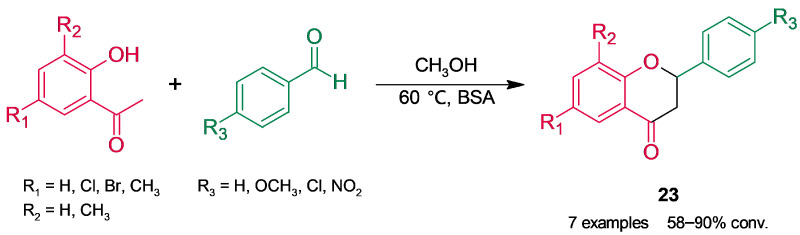
Bovine serum albumin (BSA)-catalyzed access to flavanones.

Flavanones (2-phenylchroman-4-ones), a group of flavonoids with more than 300 structures identified in nature, possess highly diverse biological activity [67]. Their conventional synthesis is based on Claisen–Schmidt condensation of a 2′-hydroxyacetophenone and an aldehyde to give a chalcone, whose subsequent cyclization through an intramolecular oxa-Michael addition affords the target flavanones (**23**, Figure 21). Since the hot and strong basic medium involved in the synthesis promotes side reactions, in our laboratory, we extended our results of BSA-catalyzed Claisen–Schmidt condensation (Section 2.1.1) and tested albumin in a tandem Claisen–Schmidt condensation–oxa-Michael addition. After a screening of reaction parameters (solvent, substrate molar ratio, and temperature), flavanones were obtained in a 58–90% conversion [68].

**Figure 22:**
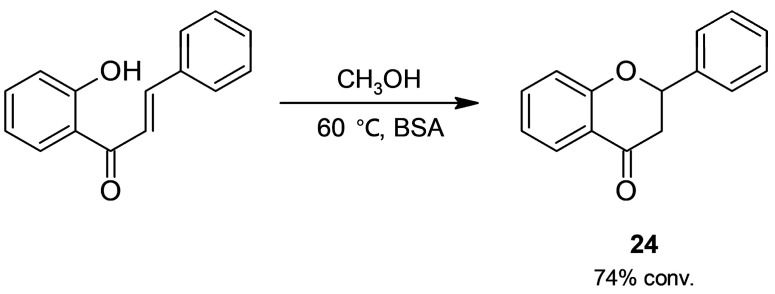
Bovine serum albumin (BSA)-catalyzed intramolecular cyclization of 2′-hydroxychalcone to flavanone.

Experiments indicated that albumin catalyzes chalcone formation and accelerates its subsequent cyclization (Figure 22), while an assay without BSA only gave low conversion. Even though BSA’s activity in Michael and thia-Michael addition has already been reported [22], its oxa-Michael activity had not been demonstrated yet.

#### 3.2.2. Nitrogenated Heterocycles

**Figure 23:**
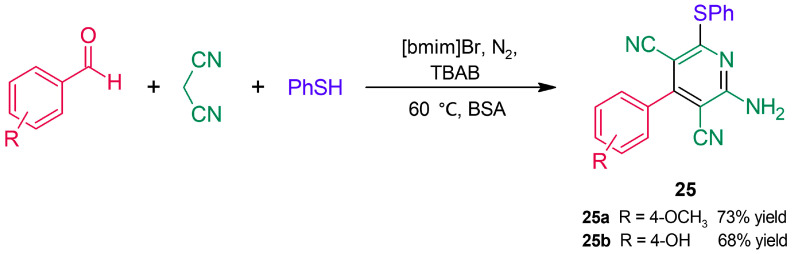
Nicotinonitriles obtained by BSA catalysis in ionic liquid.

Following their approach to obtain β-aryl-β-sulfidocarbonyl compounds (Figure 16), Sinha and coworkers [52] obtained polysubstituted pyridine derivatives through BSA catalysis (Figure 23). Instead of the expected β-sulfido compounds, products bearing the nicotinonitrile (3-cyanopyridine) core (**25**) were formed through a tandem process comprising Knoevenagel condensation–Michael addition followed by addition of the thiol to a nitrile, cyclization, tautomerization, and a final oxidation.

**Figure 24:**
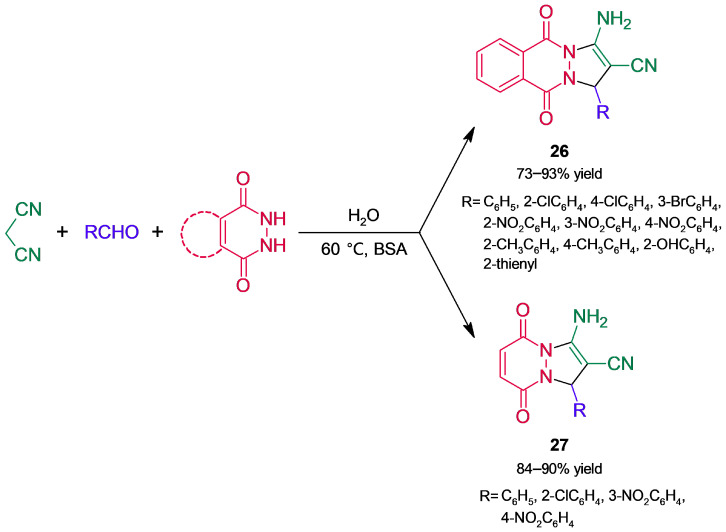
Bovine serum albumin (BSA)-catalyzed access to pyrazolophthalazine and pyrazolopyridazine derivatives.

In another application of albumin to build pyrazole core derivatives, a set of derivatives of the pyrazolophthalazine ring (1*H*-pyrazolo[1,2-b]phthalazine-5,10-diones, **26**, Figure 24) were obtained in good high yields through a BSA-catalyzed three-component reaction in water [69]. One of the substrates was a hydrazide, and the MCR involved tandem Knoevenagel condensation–Michael addition followed by cyclization and tautomerization. BSA outperformed the assayed promiscuous enzymes, and most of the tested organic solvents resulted in poor conversions, while temperature and albumin loading had less influence on the biotransformation. Replacement of phthalic hydrazide by maleic hydrazide resulted in obtaining pyrazolopyridazine derivatives (1*H*-pyrazolo[1,2-a]pyridazine-5,8-diones, **27**) in high yields.

**Figure 25:**
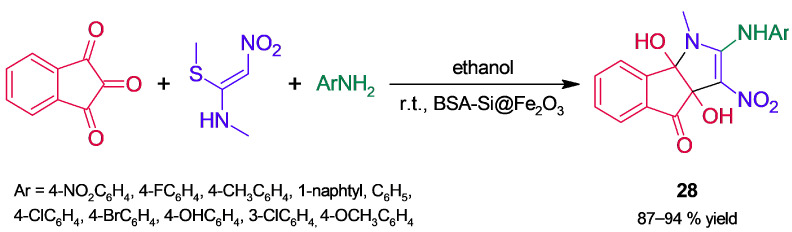
Synthesis of pyrrole derivatives through an MCR catalyzed by BSA-Si@Fe_2_O_3._

In a different catalytic approach, Siddiqi and coworkers [70] supported magnetically separable nanoparticles (NPs) of gamma-magnetite (γ-Fe_2_O_3_ NPs) on silica-functionalized albumin (BSA-Si), obtaining a hybrid catalyst (BSA-Si@Fe_2_O_3_). Its structure was characterized by a set of techniques, such as FT-IR, TEM, XRD, and EPR. BSA-Si@Fe_2_O_3_ was applied in the synthesis of pyrrole derivatives (**28**, Figure 25) through an MCR reaction between ninhydrin, an enamine, and *p*-nitroaniline; the process involved tandem nucleophilic addition of the enamine to ninhydrin, subsequent nucleophilic addition of the amine, and elimination of methanethiol. In contrast to the hybrid catalyst, an assay of BSA, BSA-Si, and γ-Fe_2_O_3_ NPs led to lower product yields; the higher performance of the hybrid was attributed to the combined effect of both NPs and BSA-Si. The study comprised optimization of the reaction solvent and catalyst loading; the procedure was extended to a set of anilines, giving pyrrole derivatives **28** in high yields and with short reaction times. The hybrid catalyst showed no significant loss of activity after six reaction cycles; assays indicated that no catalyst leaching occurred during the reaction.

**Figure 26:**
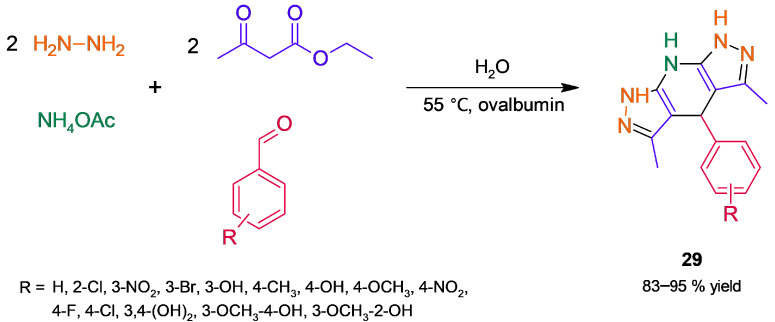
Ovalbumin-catalyzed synthesis of 1,4,7,8-tetrahydrodipyrazolopyridines.

**Figure 27:**
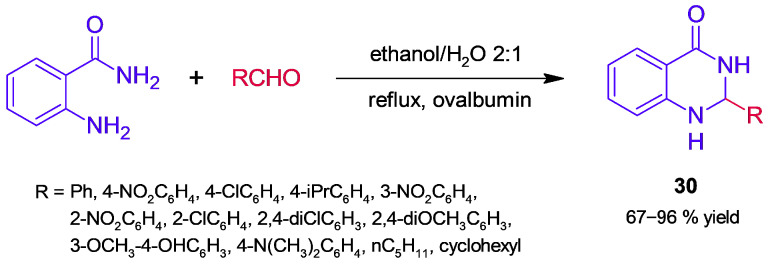
Ovalbumin-catalyzed synthesis of 2,3-dihydroquinazolin-4(1*H*)-ones.

On the other hand, ovalbumin is the main protein of egg white; it differs structurally from mammalian serum albumins since it is a globular phosphoglycoprotein of MW = 44.5 kDa [71]. Salehi and Mirjalili [72] have set up a simple protocol to separate ovalbumin in a nanometer size from egg white, characterizing it by a set of methods, such as MALDI-TOF and FESEM. The obtained nanoovalbumin was tested as a catalyst for the synthesis of dipyrazolopyridine derivatives **29** through MCR (Figure 26), resulting in a high yield of target products. Initially, condensation of ethyl acetoacetate and hydrazine affords 3-methyl-1*H*-pyrazol-5-(4*H*)-one, which subsequently reacts by aldol condensation–Michael addition, followed by the formation of an imine and cyclization tautomerization. The biocatalyzed MCR was sensitive to the reaction solvent and temperature; moreover, the biocatalyst exhibited good reusability, without significant loss of activity after four cycles. Following this approach, the synthesis of 2,3-dihydroquinazolinones (**30**, Figure 27) was achieved in moderate–high yields [73]. In this case, the formation of a Schiff base in tandem with intramolecular addition is involved. In addition, the recovered biocatalyst showed no significant loss of activity after four cycles of use.

## 4. Conclusions

This work offers a perspective on biocatalytic applications of albumin over the last decade, highlighting its potential as a green, mild, and easily available catalyst in synthetic organic chemistry. The catalytic versatility of this protein is evidenced by the wide scope of transformations in which it is active, such as additions, eliminations, and oxidations. The reviewed examples present albumin-catalyzed formation of C-C bonds by aldol condensation and Henry and Knoevenagel reactions, as well as its use for the formation of C-N and C-S bonds. Albumin offers a green procedure to convert thiols into disulfides, and its ability to act as a metalloenzyme mimic can be exploited in sulfoxidation and epoxidation reactions. In addition, this biocatalyst can be applied in tandem and multicomponent reactions, offering mild alternatives for the synthesis of oxygenated and nitrogenated heterocycles. The reviewed examples also involved water, ethanol, and ILs as reaction solvents, providing protocols in which both the catalyst and solvent are green. Overall, the perspective shows that this protein may be integrated into chemoenzymatic approaches when mildness, sustainability, and availability are required. Moreover, its versatility seems to indicate that it will find further applications in the catalysis of organic reactions. On the other hand, albumin’s excellent reusability suggests the potential of this protein from a biotechnological point of view.

## Data Availability

No new data were created or analyzed in this study.

## Abbreviations

The following abbreviations are used in this manuscript:

BHT	Butylated hydroxytoluene
DFT	Discrete Fourier transform
EPR	Electron paramagnetic resonance
ESI-MS	Electrospray ionization mass spectrometry
FESEM	Field emission scanning electron microscope
FT-IR	Fourier-transform infrared spectroscopy
MALDI-TOF	Matrix-assisted laser desorption/ionization time-of-flight
NMR	Nuclear magnetic resonance
TEM	Transmission electron microscopy
TEMPO	2,2,6,6-Tetramethylpiperidine-1-oxyl
UV–vis	Ultraviolet–visible
XRD	X-ray diffraction
